# Lymphatic contractile dysfunction in mouse models of Cantú Syndrome with K_ATP_ channel gain-of-function

**DOI:** 10.1093/function/zqad017

**Published:** 2023-04-18

**Authors:** Michael J Davis, Jorge A Castorena-Gonzalez, Hae Jin Kim, Min Li, Maria Remedi, Colin G Nichols

**Affiliations:** Department of Medical Pharmacology and Physiology, University of Missouri School of Medicine, Columbia MO 65212, USA; Department of Pharmacology, Tulane University School of Medicine, New Orleans LA 70112, USA; Department of Medical Pharmacology and Physiology, University of Missouri School of Medicine, Columbia MO 65212, USA; Department of Medical Pharmacology and Physiology, University of Missouri School of Medicine, Columbia MO 65212, USA; Center for the Investigation of Membrane Excitability Diseases, Washington University School of Medicine, St. Louis, MO 63110, USA; Division of Endocrinology, Diabetes and Metabolism, Department of Medicine, Washington University School of Medicine, St. Louis, MO 63110, USA; Center for the Investigation of Membrane Excitability Diseases, Washington University School of Medicine, St. Louis, MO 63110, USA; Department of Cell Biology and Physiology, Washington University School of Medicine, St. Louis, MO 63110, USA

**Keywords:** Kir6.1, SUR2, primary lymphedema, lymphatic pumping, smooth muscle excitability, glibenclamide

## Abstract

Cantú Syndrome (CS) is an autosomal dominant disorder caused by gain-of-function (GoF) mutations in the Kir6.1 and SUR2 subunits of K_ATP_ channels. K_ATP_ overactivity results in a chronic reduction in arterial tone and hypotension, leading to other systemic cardiovascular complications. However, the underlying mechanism of lymphedema, developed by >50% of CS patients, is unknown. We investigated whether lymphatic contractile dysfunction occurs in mice expressing CS mutations in Kir6.1 (Kir6.1[V65M]) or SUR2 (SUR2[A478V], SUR2[R1154Q]). Pressure myograph tests of contractile function of popliteal lymphatic vessels over the physiological pressure range revealed significantly impaired contractile strength and reduced frequency of spontaneous contractions at all pressures in heterozygous Kir6.1[V65M] vessels, compared to control littermates. Contractile dysfunction of intact popliteal lymphatics in vivo was confirmed using near-infrared fluorescence microscopy. Homozygous SUR2[A478V] vessels exhibited profound contractile dysfunction ex vivo, but heterozygous SUR2[A478V] vessels showed essentially normal contractile function. However, further investigation of vessels from all three GoF mouse strains revealed significant disruption in contraction wave entrainment, decreased conduction speed and distance, multiple pacemaker sites, and reversing wave direction. Tests of 2-valve lymphatic vessels forced to pump against an adverse pressure gradient revealed that all CS-associated genotypes were essentially incapable of pumping under an imposed outflow load. Our results show that varying degrees of lymphatic contractile dysfunction occur in proportion to the degree of molecular GoF in Kir6.1 or SUR2. This is the first example of lymphatic contractile dysfunction caused by a smooth muscle ion channel mutation and potentially explains the susceptibility of CS patients to lymphedema.

## Graphical Abstract

**Graphical Abstract:**
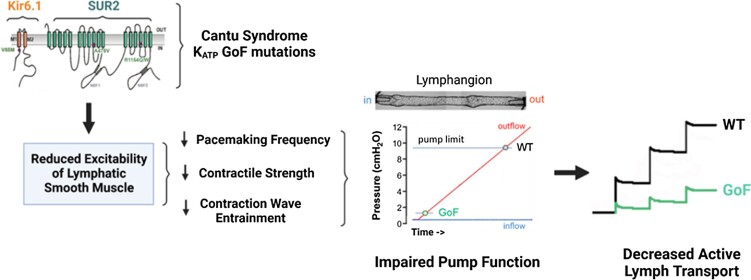

Significance Statement Contractile function studies in lymphatic vessels from mice with gain-of-function mutations in the Kir6.1 and SUR2 subunits of K_ATP_ channels, replicating mutations that occur in human Cantú Syndrome, exhibit weaker contractile strength, reduced frequency of spontaneous contractions and loss of contraction wave entrainment, resulting in severely reduced lymphatic pump strength. These findings are the first example of lymphatic contractile dysfunction caused by a smooth muscle ion channel mutation and potentially explain the susceptibility of Cantú Syndrome patients to lymphedema.

## Introduction

ATP-sensitive potassium (K_ATP_) channels, activated by intracellular ADP and inhibited by intracellular ATP, are present in many tissues, coupling the metabolic state of the cell to electrical activity.^1^ The channels are formed as octameric complexes of pore-forming (Kir6) and modulatory sulphonylurea receptor (SUR) subunits, encoded by two Kir6 genes, *KCNJ8* (Kir6.1) and *KCNJ11* (Kir6.2), and two SUR genes, *ABCC8* (SUR1) and *ABCC9* (SUR2). Alternate splicing of *ABCC9* produces two protein variants, SUR2A and SUR2B, that differ in their C-terminal sequences.^2,3^ The expression of different combinations of Kir6 and SUR subunit genes in different cell types results in tissue-specific K_ATP_ channel properties and functional roles. For example, Kir6.2/SUR1 channels regulate insulin secretion by pancreatic β-cells,^4^ Kir6.2/SUR2A channels modulate action potential duration during metabolic stress in ventricular cardiomyocytes, and Kir6.1/SUR2B channels control basal tone development by arterial smooth muscle cells.^3^

Gain-of-function (GoF) mutations in the muscle-type Kir6.1 and SUR2 subunits of K_ATP_ channels are associated with an autosomal dominant disorder, Cantú Syndrome (CS).^5,6^ Over 30 mutations in *KCNJ8* and *ABCC9* have now been linked to CS.^6–8^ CS subjects are characterized by a complex constellation of symptoms, with almost all subjects exhibiting cardiovascular abnormalities, including hypotension, cardiomegaly, and/or pulmonary hypertension.^6,7,9,10^ Systemic blood pressure^11^ is chronically reduced due to a loss of tone in arterial smooth muscle, which leads to a reflex increase in sympathetic nerve outflow to the heart and activation of the renin-angiotensin system, eventually producing ventricular hypertrophy and a high output state associated with cardiac failure.^10,12,13^ Many, if not most, CS subjects eventually develop marked lymphedema, primarily in dependent extremities,^6,14^ but also potentially linked to their distinct facial features.

The underlying mechanisms of lymphedema in CS patients are unknown, but the loss of contractile function in arteries/arterioles predicts a similar loss of contractile function in lymphatic vessels, given that smooth muscle cells in both types of vessels express Kir6.1 and SUR2B.^15^ The spontaneous contractions of lymphatic smooth muscle provide a propulsive force to move lymph centrally and to maintain tissue interstitial fluid balance.^16^ Active lymphatic contractions are initiated by action potentials in lymphatic smooth muscle cells (LMCs) that result from interactions of voltage-dependent Na^+^, Ca^2+^, K^+^, and Cl^−^ channels,^17–24^ which produce depolarizing pacemaking potentials and action potentials. The K_ATP_ channels expressed in these cells appear to exert a tonic hyperpolarizing action and inhibitory influence on excitability,^25^ based on the observations that (1) the K_ATP_ channel inhibitor glibenclamide depolarizes lymphatic muscle^26,27^ and increases the frequency of spontaneous lymphatic contractions;^28^ and (2) K_ATP_ channel openers hyperpolarize lymphatic muscle^15,26^ and inhibit active lymphatic pumping.^28–30^ We hypothesized that chronically hyperactive K_ATP_ channels in LMCs would retard the generation of spontaneous action potentials and lymphatic contractions. Because lymph flow normally occurs against a hydrostatic pressure gradient from peripheral tissues to the central venous system,^16^ and this gradient is exacerbated in dependent extremities under a gravitational load,^31^ any impairment of active lymphatic contractile function would substantially impact the return of lymph from the periphery and promote fluid and protein retention. The only current treatment options for lymphedema, including for CS-related lymphedema, are palliative measures such as external compression and massage. Thus, understanding the contributions of K_ATP_ channels to lymphedema in CS patients would facilitate the development of pharmacologic strategies to treat the underlying K_ATP_ channel hyperactivity.

We recently reported that expression of a GoF transgene in smooth muscle, consisting of an activating double mutation in Kir6.1, results in profound contractile dysfunction of mouse collecting lymphatics^15^: Expression of Kir6.1[GD-QR] channels under control of the *Myh11*-CreER^T2^ promoter leads to LMC hyperpolarization, cessation of lymphatic action potentials, and impairment of spontaneous lymphatic contractions. Importantly, *Myh11*-CreER^T2^; *Kir6.1[GD-QR]^f/f^* mice do not reiterate the global consequences of mutations in endogenous genes and hence cannot fully model CS, nor can they predict the severity of excitability changes that will result from such mutations. Therefore, in the present study we investigated lymphatic function in recently developed “Cantú” mouse models, in which CS-associated mutations in Kir6.1 (V65M) or in SUR2 (A478V or R1154Q), were introduced into the respective endogenous murine loci using CRISPR/Cas9 engineering. The systemic consequences of these mutations for cardiovascular,^11,12^,^32–34^ skeletal muscle,^35,36^ and gastrointestinal^37^ function have been recently investigated. Mirroring what is observed in CS patients, these mice exhibit dramatically enlarged hearts with increased ejection fraction and increased contractility. Systemic arteries are dilated and arterial compliance is increased, leading to chronically reduced blood pressure.^11^ The severity of cardiovascular symptoms in mice with CS-associated mutations generally increases with the severity of K_ATP_ channel hyperactivity, when the latter is assessed by electrophysiological studies of isolated vascular smooth muscle cells.^11^ The Kir6.1[V65M] mutation has a more marked effect than the SUR2[A478V] mutation and homozygous animals are more severely affected than heterozygous animals in each case.^11^ The most severely affected homozygous V65M animals (*Kir6.1^VM/VM^*) typically die very young. This early death is particularly prominent shortly after weaning, and potentially reflects gastro-intestinal complications, since it is partially avoided by liquid diet.^12,37^ The most common (>30%) CS-associated variants result in mutation R1154Q (or less commonly R1154W) at a single residue in SUR2. A third mouse, in which this variant was introduced to the *ABCC9* locus by CRISPR mutation, also resulted in CS-like symptoms, but these were very mild, in this case due to an additional alternate splicing consequence that results in a decrease in the number of functional channels,^34^ but which appears to not occur in human tissues.

Given the prior findings with transgenic K_ATP_ GOF animals, and the prominence of Kir6.1 and SUR2 in lymphatic smooth muscle, we sought to understand the extent to which lymphatic contractile function might be compromised in mice with CS-associated mutations, with the potential to explain the susceptibility of CS patients to lymphedema, using both ex vivo and in vivo assays in heterozygous *Kir6.1[V65M]*, hetero- and homozygous *SUR2[A478V]* mice and, for completeness, in homozygous *SUR2[R1154Q]* mice.

## Materials and Methods

### Mice

All procedures were reviewed and approved by the University of Missouri Animal Care and Use Committee and complied with the standards stated in the “Guide for the Care and Use of Laboratory Animals” (National Institutes of Health, revised 2011). The generation of *Kir6.1[V65M], SUR2[A478V]* and *SUR2[R1154Q]* mice was described previously.^11^ For smooth muscle-specific genetic deletion of the L-type calcium channel (Cav1.2), *Myh11*-CreER^T2^ mice (B6.FVB-Tg(*Myh11*-cre/ERT2)1Soff/J) obtained from Stefan Offermanns (Max-Planck Institute, Bad Neuheim) were bred with *Ca_v_1.2^f/f^* mice (*Cacna1c*^tm3Hfm^/J; #024 714), purchased from JAX, to generate *Myh11*-CreER^T2^*; Ca_v_1.2^f/f^* (*Cav1.2*^SM-KO^)mice. The offspring were injected with tamoxifen (10 mg/ml, 100ul i.p.) for 5 consecutive days and allowed to recover for 10–12 days before testing, as previously described.^38^ The genotyping of *Kir6.1[V65M], SUR2[A478V]* and *SUR2[R1154Q]* mice, subsequently referred to as *Kir6.1^wt/^^VM^, SUR2^AV/AV^* or *SUR2^wt/AV^*, and *SUR2^RQ/RQ^* mice, respectively, was performed by Transnetyx (Cordova, TN). For genotyping of other mice, genomic DNA was extracted from tail clips using the HotSHOT method and genotypes were determined by PCR with 2x PCR Super Master Polymerase Mix (Catalog # B46019, Bimake, Houston, TX) according to the provider's instructions. Mice were provided *ad libitum* access to food and water and housed under normal light and dark cycles in cages of up to five mice. Mice of both sexes were used for these experiments, except for protocols on *Myh11*-CreER^T2^*; Ca_v_1.2^f/f^* mice in which the Cre is carried on the Y chromosome.

### Vessel Isolation, Pressure Myograph, and Data Acquisition

Mice were anesthetized by intraperitoneal injection of either pentobarbital-sodium (60 mg/kg) or Ketamine/Xylazine (100/10 mg/kg) and placed in the prone position on a heated tissue dissection/isolation pad. The superficial saphenous vein was exposed by a proximal-to-distal incision in the skin over the calf and the popliteal afferent lymphatic vessels on each side of the vein were identified and isolated as previously described.^39^ Each vessel was then pinned with short segments of 40 µm stainless steel wire onto a Sylgard-coated dissection chamber filled with BSA-containing Krebs buffer at room temperature. The surrounding adipose and connective tissues were removed by microdissection and the vessel was transferred to a 3-mL observation chamber on the stage of a Leica inverted microscope, where it was cannulated and pressurized to 3 cmH_2_O using two glass micropipettes (50 to 60 µm outside diameter). While pressurized, the segment was cleared of remaining connective and adipose tissue. Polyethylene tubing attached to the back of each glass micropipette connected the vessel to a computerized pressure controller^40^ for independent control of inflow pressure (Pin) and outflow pressure (Pout). To minimize diameter-tracking artifacts associated with longitudinal bowing of the vessel at higher intraluminal pressures, input and output pressures were briefly set to 10 cmH_2_O at the beginning of every experiment, during which time the segment was stretched axially to remove any longitudinal slack.

### Assessment of Contractile Function and Response to Pressure

The contractile parameters of each vessel were characterized at intraluminal pressures spanning the physiological range from 0.5 to 10 cmH_2_O (in successive steps from 3 to 2, 1, 0.5, 3, 5, 8, and 10 cmH_2_O). Spontaneous contractions were recorded at each pressure step in typical intervals of 2 to 5 min. Input and output pressures were maintained at equal levels so that there was no imposed pressure gradient for forward flow. After testing the pressure responses of a vessel in Krebs buffer, glibenclamide (10 μm) was added to the perfusate for 2 to 5 min and the pressure steps were repeated in its continued presence. At the end of each experiment, the vessel was equilibrated by perfusion with calcium-free Krebs buffer containing 3 mM EGTA for 30 min, and passive diameters were obtained at each level of intraluminal pressure.

### Contractile Function Parameters

Internal diameter was tracked continuously throughout the experiment.^41^ Afterward, custom LabVIEW programs detected end diastolic diameter (EDD), end systolic diameter (ESD), and contraction frequency (FREQ) on a contraction-by-contraction basis. Each parameter was averaged over a 2 to 5 min period and used to calculate the following indices of lymphatic contractile function:


(1) $$\begin{equation*}
{\rm{Contraction\ Amplitude\ (AMP)\ = \ EDD - ESD}}
,\end{equation*}$$

(2) $$\begin{equation*}
{\rm{Normalized\ Contraction\ Amplitude\ = \ }}\left( {\frac{{{\rm{EDD - ESD}}}}{{{{\rm{D}}}_{{\rm{MAX}}}}}} \right){\rm{ \times 100}}
,\end{equation*}$$

(3) $$\begin{equation*}
{\rm{Ejection\ Fraction\ (EF)\ = \ }}\left[ {\frac{{{\rm{ED}}{{\rm{D}}}^{\rm{2}}{\rm{ - ES}}{{\rm{D}}}^{\rm{2}}}}{{{\rm{ED}}{{\rm{D}}}^{\rm{2}}}}} \right]
,\end{equation*}$$

(4) $$\begin{equation*}
{\rm{Fractional\ Pump\ Flow\ (FPF)\ = \ EF}} \cdot {\rm{FREQ}}
,\end{equation*}$$

(5) $$\begin{equation*}
{\rm{Tone\ = \ }}\left( {\frac{{{{\rm{D}}}_{{\rm{MAX}}}{\rm{ - EDD}}}}{{{{\rm{D}}}_{{\rm{MAX}}}}}} \right){\rm{ \times 100}}
,\end{equation*}$$

where D_MAX_ represents the maximum passive diameter (obtained after incubation with calcium-free Krebs solution) at a given level of intraluminal pressure.

### Pump Tests

To assess the impact of Cantu mutations on the pumping ability of single, 2-valve lymphangions, we conducted a second ex vivo protocol, similar to that described previously.^31,42,43^ With Pin and Pout set at or below 3 cmH_2_O, Pout was elevated ramp-wise to 10–12 cmH_2_O at a rate of ∼3 cmH_2_O/min, with Pin held constant and while monitoring the position of the outflow valve. Successful ejection during a contraction cycle was associated with opening of the outflow valve as the peak of the internal pressure spike associated with contraction transiently exceeded Pout;^44^ when the peak did not exceed Pout, the outflow valve failed to open during lymphatic systole. Eventually the pump weakened and the Pout value (minus Pin) at the time of the last successful ejection corresponded to the “pump limit” of the lymphangion. Close agreement between the output valve position and the peak of the systolic internal pressure spike (as measured using a servo-nulling micropipette) was demonstrated previously,^31,43^ so in the present study it was not necessary to measure Psn. Valve position, either open or closed, was determined from replay of the recorded protocol videos using a LabVIEW program, as described previously,^42^ while maintaining synchronization of the valve position data with the pressure and diameter data. Typically, three identical Pout ramps were performed, several minutes apart, and the average value of the pump limit, defined as ΔP (Pout—Pin) at the last successful ejection, was determined. Because the optimal preload for an individual vessel could vary from 1 to 3 cmH_2_O, the 3-ramp protocol was determined at Pin values of 1, 2, and 3 cmH_2_O, if possible.

A variation of this protocol was needed in instances in which the outflow valve “locked” open in diastole during a Pout ramp, a behavior shown to occur ∼50% of the time if the inflow valve has a lower ΔP (Pout—Pin) for passive closure than the outflow valve at any particular pressure.^44,45^ To circumvent this problem in the subset of vessels exhibiting the behavior, we used an alternate protocol. When the outflow valve had transiently closed during lymphatic diastole at equal Pin and Pout, due to the suction created by vessel wall expansion,^46^ Pout was stepped rapidly to 12 cmH_2_O, forcing the outflow valve to remain closed. Pout was then lowered, ramp-wise, at ∼3 cmH_2_O/min while spontaneous contractions continued to occur. As Pout declined, the outflow valve first opened during lymphatic systole at the point corresponding to the pump limit (as well as in subsequent contractions). We confirmed in another publication that the pump limit determined by this protocol was very close (within 0.3 cmH_2_O) to that determined by the ascending Pout ramp protocol.^47^ In some vessels, the inflow valve closed before the outflow valve in response to both the ascending Pout ramp and the rapid Pout step so that a pump test could not be performed.

### Contraction Wave Analysis

To quantify the degree of entrainment of contraction waves, brightfield videos of spontaneous contractions were acquired for 3 to 4 min at video rates ranging from 30 to 50 fps. Recorded videos were then stored for offline processing, analysis, and quantification of the conduction speed. Videos of contractions were processed frame by frame to generate two-dimensional spatiotemporal maps (STMs) representing the measurement of the outside diameter (encoded in 8-bit grayscale) over time (horizontal axis) at every position along the vessel (vertical axis), as described previously.^31^ All video processing and analyses were performed using a set of custom-written Python programs. Conduction speed was determined for each wave by the slope of the corresponding band on the ST map (by linear fit of the points defining the leading edge) and the speeds were averaged for all the contractions in a given video. An “entrainment index “ for each vessel was calculated as the % of contraction waves that conducted over the entire length of the vessel.

### NIRF Imaging of Lymphatic Transport Under a Gravitational Load

Dye transport through the lymphatic system was assessed under conditions where transport was accomplished primarily by active lymphatic contractions rather than passive movement, tissue compression, or artificial hydrostatic gradients created by tracer injection. A mouse was anesthetized with isofluorane (3% for induction, 1.8% for maintenance) and the hair removed from both hindlimbs using a depilatory cream. After thorough rinsing and drying of the skin, the mouse was placed on a heated platform (37°C) in an enclosed, temperature-controlled, light-proof box. Fluorescence images of the popliteal region through the intact skin were collected using a Leica S6 macroscope (NCI, Minneapolis MN) coupled to a Hamamatsu Orca ER camera, as described previously.^31^ LabVIEW programs were used to control the camera, transfer the images to a Windows 7 computer and save images to disk, at 1 frame per second over a time period of up to 1 h. The field of view, 2.5 × 2.0 mm, was illuminated by a 1-watt 785 nm laser and aspheric lens (Laserglow, Toronto, Canada) through a 785 ± 5 nm clean-up excitation filter (Chroma, Bellows Falls, VT), and the fluorescence signal was collected through 795 nm high pass dichroic and 820 ± 20 emission filters (Chroma). The entire apparatus was mounted on a tilt-table that allowed the mouse hindlimb to be imaged in either a horizontal or near-vertical position (12° from vertical). The latter body position resulted in the imposition of ∼5 cmH_2_O hydrostatic load on the hindlimb of the adult animal due to gravitational forces acting on the column of lymph between the lower leg and the neck. To prevent shifts in the hindlimb position under these conditions the mouse was fitted with an elastic cloth harness around its thorax, which was secured to the microscope stage, thus supporting the animal's weight. The mouse remained in this position for at least 10 min prior to dye injection and imaging.

A small volume (1 µL) of IRDye^®^ 800CW PEG Contrast Agent (LiCor, Lincoln, NE), dissolved in sterile saline (10 nmol/100 µL), was injected intradermally into the dorsal surface of the foot. Injection of a minimal volume of tracer with the animal in the near-vertical position was sufficient to allow imaging of one or both of the two main popliteal lymphatic afferent vessels running from the foot to the ankle and then to the popliteal node, while increasing the likelihood that the only transport observed under those conditions was pulsatile, i.e., occurring in “dye packets,” as described in previous publications.^48,49^ To maximize consistency, injections were made between two tributaries of the saphenous vein, within ∼1 mm^2^ area on the dorsal surface of the foot, using a 200-μm 34 ga needle. Subsequent analysis of the captured videos allowed us to determine the time elapsed from injection of the dye to its first appearance in a popliteal collector, which typically ranged from 2 to 20 min. The variability presumably resulted from differences in the proximity of the injection site to a nearby collector in the foot (which was dictated by the network anatomy of individual animals), in addition to a variable degree of active pumping of the collecting vessel(s) in that limb. In some cases, dye appearance in one or more vessels occurred within 2 s after injection, presumably indicative of direct injection into a collector in the foot. Such cases were considered artifactual and were excluded from the analysis. Once the dye wavefront appeared in a popliteal collector, the path length of the wavefront along the vessel and the number of elapsed frames needed for transit of that path were determined using ImageJ; these parameters were used to calculate the wavefront speed. A third parameter, packet frequency, was determined as in previous publications,^48,50,51^ from the frequency of the oscillations in the intensity of dye signal within a filled vessel (the average within 3 regions of interest), which corresponded to active contractions that generated propulsive lymph movement. A minimum 3% change in signal intensity within a 20-sec time period was considered to represent a single packet. In cases where no dye front appeared within 50 min, (e.g., some of the *Cav1.2^SM-KO^* studies) we confirmed successful dye injection by gently pressing on the skin over the injection site with a cotton swab and in all cases dye rapidly appeared in the downstream popliteal network within the imaging window.

### Solutions and Chemicals

Krebs buffer contained: 146.9 mM NaCl, 4.7 mM KCl, 2 mM CaCl_2_ 2H_2_O, 1.2 mM MgSO_4_, 1.2 mM NaH_2_PO_4_ H_2_O, 3 mM NaHCO_3_, 1.5 mM Na-HEPES, and 5 mM D-glucose (pH = 7.4). An identical buffer (“Krebs-BSA”) also contained 0.5% bovine serum albumin. Krebs-BSA buffer was present both luminally and abluminally during cannulation, but the abluminal solution was constantly exchanged with plain Krebs during the experimental protocol. For Ca^2+^-free Krebs, 3 mM EGTA replaced CaCl_2_ 2H_2_O. All chemicals were obtained from Sigma–Aldrich (St. Louis, MO, USA) except BSA (US Biochemicals; Cleveland, OH, USA), MgSO_4_ and Na-HEPES (ThermoFisher Scientific; Pittsburgh, PA, USA). Glibenclamide (GLIB, Sigma–Aldrich) was prepared in DMSO as a stock solution and then diluted in Krebs solution to reach the final concentration stated.

### Statistical Tests

**Figure 1.**
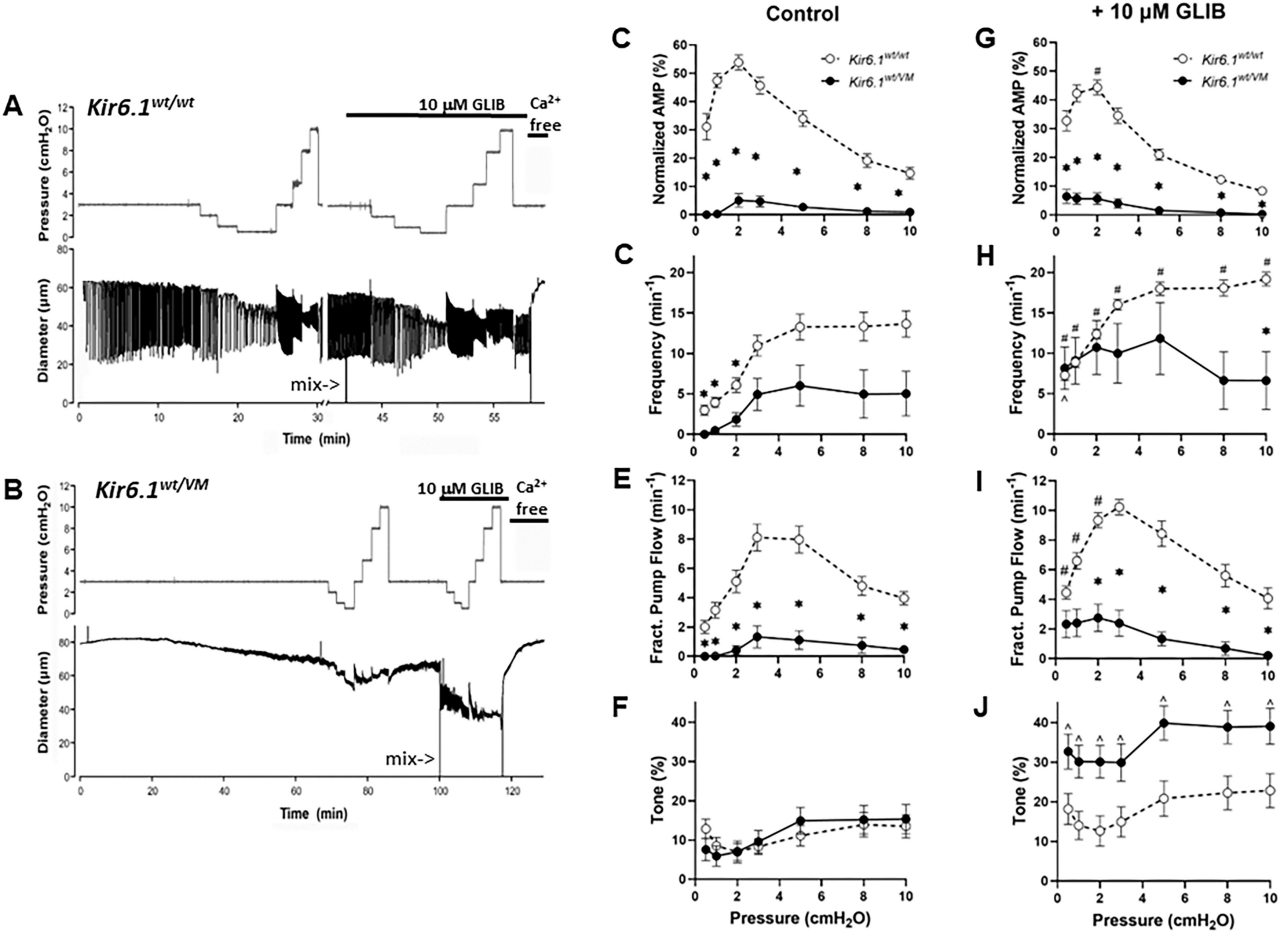
Lymphatic contractility is impaired in *Kir6.1^wt/^^VM^* mice. Recordings of spontaneous contractions in popliteal lymphatic vessels isolated from (A) a *Kir6.1^wt/^^wt^* littermate control mouse and (B) a *Kir6.1^wt/^^VM^* heterozygous mouse. Each vertical line in the diameter trace represents a single contraction lasting 1-2 sec. The time required to develop spontaneous contractile activity was less than a few min in the *Kir6.1^wt/^^wt^* vessel (typical of control vessels of all genotypes), but ∼40 min in the *Kir6.1^wt/^^VM^* vessel. The *Kir6.1^wt/^^wt^* vessel developed large, propulsive contractions that changed in both frequency and amplitude as a function of pressure, whereas the *Kir6.1^wt/^^VM^* vessel developed only high frequency small amplitude diameter oscillations that were largely independent of pressure. (C to J) Summary of contractile properties for popliteal lymphatics from *Kir6.1^wt/^^VM^* heterozygous mice (*n* = 11) and their *Kir6.1^wt/^^wt^* littermate controls (*n* = 7) in the absence (C to F) or presence (G to J) of 10 μm GLIB. *Significant difference between *Kir6.1^wt/^^wt^* and *Kir6.1^wt/^^VM^ vessels* in the absence or presence of 10 μm GLIB (*P* < 0.05). #Significant difference between *Kir6.1^wt/^^wt^* vessels in presence vs. absence of GLIB (*P* < 0.05). ^Significant difference between *Kir6.1^wt/^^VM^* vessels in presence vs. absence of GLIB (*P* < 0.05).

**Figure 2.**
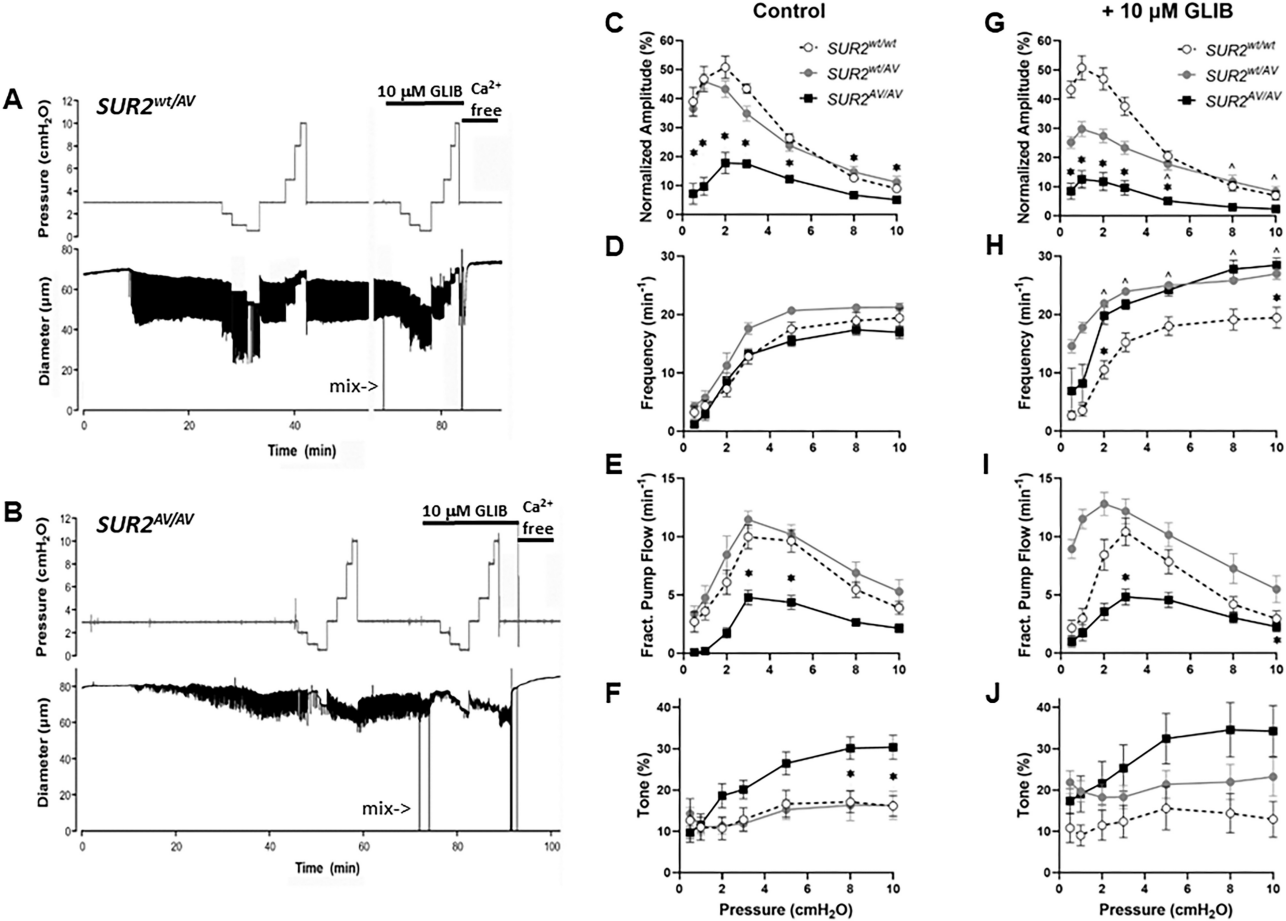
Lymphatic contractility is impaired in *SUR2^AV/AV^* mice. Recordings of spontaneous contractions in popliteal lymphatic vessels isolated from (A) a *SUR2^wt/AV^* heterozygous mouse and (B) a *SUR2^AV/AV^* homozygous mouse. Spontaneous contractions developed in both vessels and were of nearly normal amplitude in the *SUR2^wt/AV^* vessel but blunted in the *SUR2^AV/AV^* vessel. GLIB slightly increased contraction frequency and slightly reduced contraction amplitude in the *SUR2^wt/AV^* vessel, but severely reduced the amplitude in the *SUR2^AV/AV^* vessel. (C to J) Summary of contractile properties for popliteal lymphatics from *SUR2^wt/AV^* heterozygous mice (*n* = 7), *SUR2^AV/AV^* homozygous mice (*n* = 12), and their *SUR2^wt/wt^* (*n* = 5) littermate controls in the absence (C to F) or presence (G to J) of 10 μm GLIB. *Significant difference between *SUR2^wt/wt^* and *SUR2^AV/AV^ vessels* in the absence or presence of 10 μm GLIB (*P* < 0.05). #Significant difference between *SUR2^wt/wt^* vessels in presence vs. absence of GLIB (*P* < 0.05). ^Significant difference between *SUR2^AV/AV^* vessels in presence vs. absence of GLIB (*P* < 0.05).

**Figure 3.**
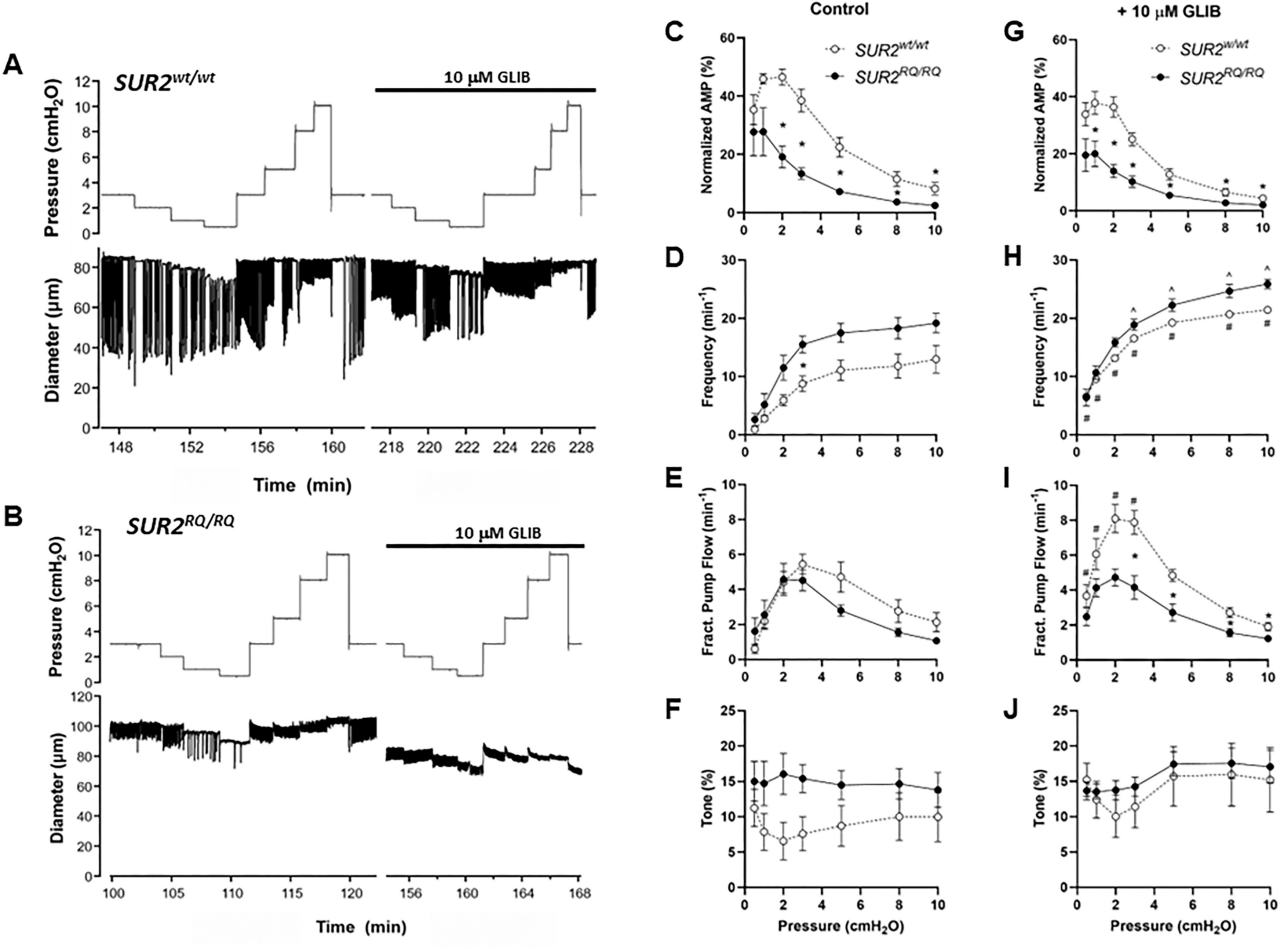
Lymphatic contractility is impaired in *SUR2^RQ/RQ^* mice. Recordings of spontaneous contractions in popliteal lymphatic vessels isolated from (A) a *SUR2^wt/wt^* mouse and (B) a *SUR2^RQ/RQ^* mouse. Summary of contractile properties for popliteal lymphatics from *SUR2^RQ/RQ^* homozygous mice (*n* = 7) and their *SUR2^wt/wt^* littermate controls (*n* = 8). *Significant difference between *SUR2^wt/wt^* and *SUR2^RQ/RQ^ vessels* in the absence or presence of 10 μm GLIB (*P* < 0.05). #Significant difference between *SUR2^wt/wt^* vessels in presence vs. absence of GLIB (*P* < 0.05). ^Significant difference between *SUR2^RQ/RQ^* vessels in presence vs. absence of GLIB (*P* < 0.05).

**Figure 4.**
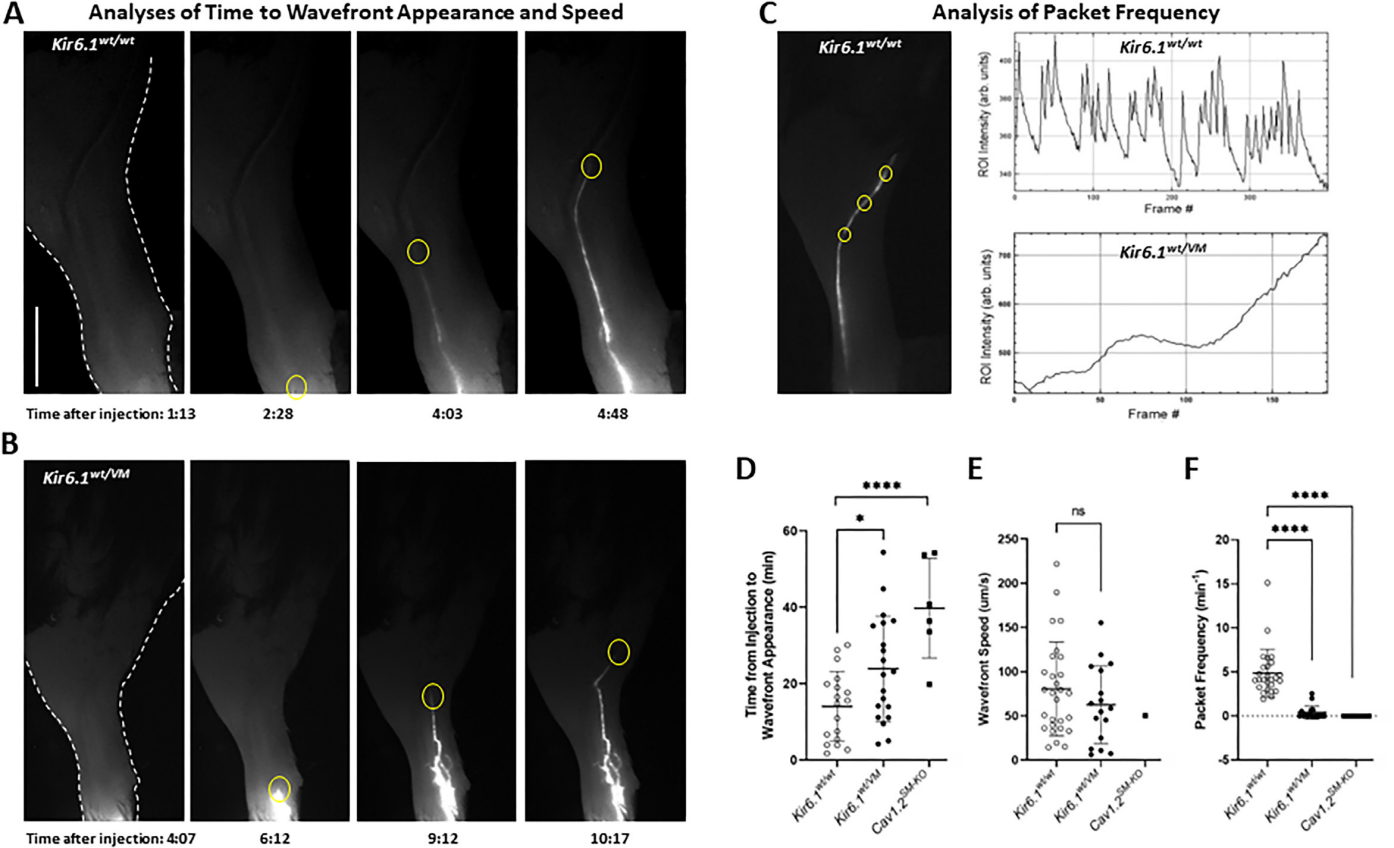
In vivo transport of IR dye in popliteal lymphatics from *Kir6.1^wt/^^wt^, Kir6.1^wt/^^VM^* and SM-specific *Cav1.2*-deficient mice. (A) Fluorescence images of the ventral surface of the left leg. The dye injection site is in the top of the foot, out of the field of view. The heel is the notch in the leg border at the lower right of image 1; the popliteal node is just out of the field of view at the top right. The background is adjusted (equally in all images) to enhance the leg borders (outlined in the left image with dotted white lines) and the saphenous vein, which is the darker line just to the left of the dye path in images 3 to 4. Calibration bar = 0.1 mm. Time units are min: sec. (B) Appearance and progression of a dye front in the left leg of a *Kir6.1^wt/^^VM^* mouse; note the relatively delayed onset and wavefront speed compared to the *Kir6.1^wt/^^wt^* littermate. (C) Packet analysis showing large, regular fluctuations in dye intensity in popliteal lymphatics of a *Kir6.1^wt/^^wt^* mouse and absence of same in popliteal lymphatics of a *Kir6.1^wt/^^VM^* mouse. (D to F) Summary of three different transport properties from *Kir6.1^wt/^^wt^* (*n* = 18), *Kir6.1^wt/^^VM^* (*n* = 20), and SM-specific *Cav1.2*-deficient mice (*n* = 6). The latter genotype was used as a reference for complete lack of active lymphatic contractions. Wavefront speed could only be reliably measured in one *Myh11CreER^T2^; Cav1.2^f/f^* mouse.

**Figure 5.**
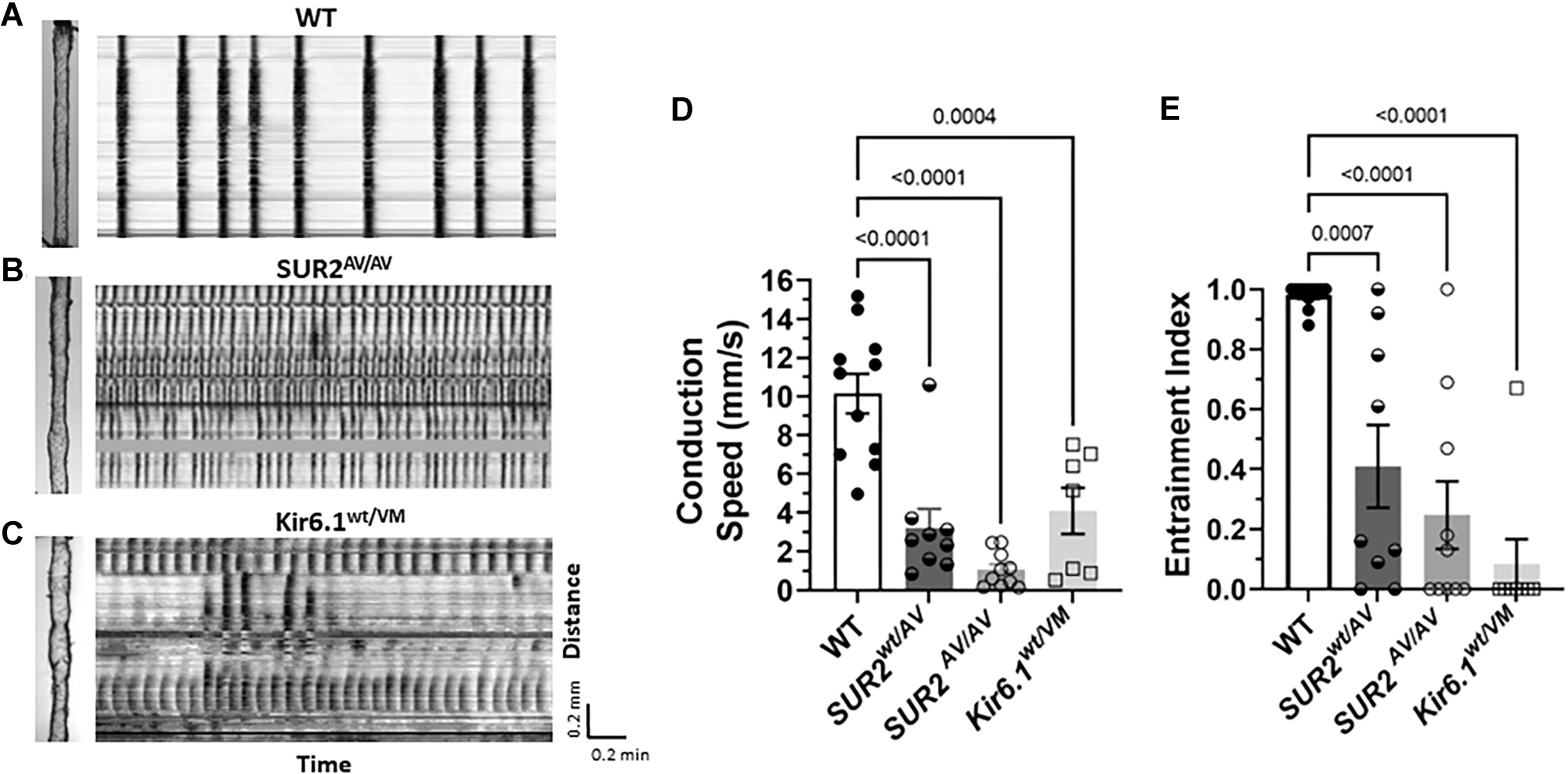
The expression of GoF K_ATP_ channels impairs conduction wave speed and entrainment. Spatio-temporal maps of spontaneous contraction waves in popliteal lymphatics from a WT (A), a *SUR2^AV/AV^* (B), and a *Kir6.1^wt/^^VM^* (C) mice. The *Y*-axis represents the distance along the vessel and corresponds to the image at the left. The *X*-axis represents time. Each vertical bar is a single contraction and the continuity of the bar, from top to bottom, indicates an entrained contraction that conducts over the entire length of the vessel. Conduction speed is given by the slope of the left edge of the band and the sign of the slope indicates the direction of the wave. The intensity of the band is inversely proportional to the degree of contraction. Horizontal bands are artifacts of diameter tracking, usually due to remaining connective tissue or fat on a localized region of the vessel. The solid gray band in the lower ST map is an example of a tracking artifact. (A) All nine of the contractions of the WT vessel are fully entrained and conduct over the entire length of the vessel. The conduction speed is too fast to resolve the direction at this timescale. (B and C) Only a few of the contractions of the *SUR2^AV/AV^* and *Kir6.1^wt/^^VM^* vessels are entrained and conduct over the entire vessel length. Most contraction waves conduct only 1/3 to 1/2 the vessel length, originate at multiple sites along the vessel and have visibly reduced conduction speed. The waves often conduct in alternating directions and in some cases change direction. (D and E) Summary of conduction speeds (D) and entrainment index (E) for WT, *SUR2^wt/AV^, SUR2^AV/AV^*, and *Kir6.1^wt/^^VM^* vessels; WT represents a combination of *SUR2^wt/wt^* and *Kir6.1^wt/^^wt^* vessels. Contraction amplitudes for 4 of 8 *Kir6.1^wt/^^VM^* vessels were too small for valid STM analyses and the data shown represent only the four vessels with sufficiently large amplitudes.

**Figure 6.**
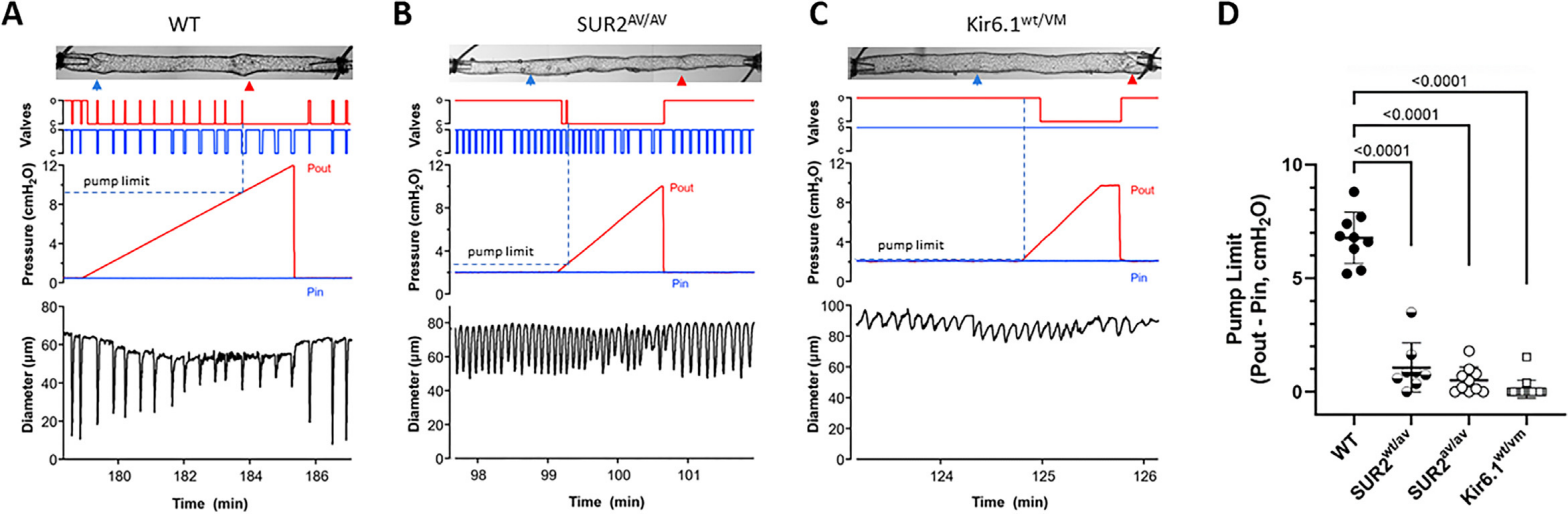
Lymphatic vessels expressing GoF K_ATP_ channels have reduced pump strength. (A to C) Examples of pump tests in (A) WT, (B) *SUR2^AV/AV^*, and *Kir6.1^wt/^^VM^* (C) lymphatic vessels, each containing two valves. Images of the vessels are shown at the top with valves indicated by arrowheads. (A) The pump limit was determined from monitoring the positions of the outflow valve (o = open; c = closed) during a Pout ramp from a low pressure to ∼10 cmH_2_O while holding Pin constant. After Pout exceeded Pin, the outflow valve was closed throughout the lymphatic contraction cycle except for a brief period of opening at the peak of lymphatic systole. When Pout exceeded the pump limit the valve failed to open. The Pout level at the peak of the previous successful ejection (marked by horizontal dotted line), minus Pin, was taken to be the pump limit. (B and C) Much lower pump limits were determined for the *SUR2^AV/AV^* and *Kir6.1^wt/^^VM^* vessels. Pin for these vessels was set to 2 cmH_2_O because no contractions were observed at lower pressures. (D) Summary data showing significantly lower average pump limits for K_ATP_ GoF vessels compared to WT vessels.

The number *n* refers to the number of vessels included per group. Statistical differences between the various parameters were assessed via 1) mixed model ANOVAs (allowing for missing values or unequal groups) with Tukey's multiple comparison tests for normally distributed data sets (Figures 1–3, 5, or 2) Mann-Whitney tests for data sets that were not normally distributed (Figures 4 and 6). Statistical analyses were performed using Prism9 (Graphpad). Data are plotted as mean ± SEM. Unless otherwise stated, the significance level was *P* < 0.05.

## Results

First, we compared lymphatic function in popliteal lymphatics from WT mice and mice carrying the CS-associated V65M mutation in the Kir6.1 subunit. After cannulation, pressurization, and equilibration to 37°C in Krebs solution, spontaneous contractions typically developed in control vessels within <10 min. Once contractions began, their amplitude and frequency stabilized after an additional ∼10 min. This pattern is illustrated by a representative diameter recording from a popliteal lymphatic vessel isolated from a *Kir6.1^wt/^^wt^* littermate mouse (Figure 1A). Changing pressure resulted in the typical pattern of response reported in previous studies,^52,53^ in which frequency declined to a minimum as pressure was lowered to 0.5 cmH_2_O and rose to a plateau value at ∼5 cmH_2_O. Contraction amplitude was maximal at 1 cmH_2_O and declined at both lower and higher pressures. Ten micromolar GLIB modestly increased contraction frequency and reduced contraction amplitude at most pressures. In contrast, robust spontaneous contractions never developed in a popliteal lymphatic vessel isolated from a *Kir6.1^wt/^^VM^* mouse, although tone began to develop at ∼30 min after equilibration at 37°C and high frequency, but very low amplitude, diameter oscillations developed after ∼40 min (Figure 1B). Unlike WT control vessels, neither amplitude nor frequency was substantially modulated by pressure. In the presence of 10 μm GLIB, the diameter oscillations of the *Kir6.1^wt/^^VM^* vessel increased slightly in amplitude and frequency, but GLIB did not noticeably affect contraction amplitude.

The contractile properties of popliteal lymphatics from *Kir6.1^wt/^^VM^* heterozygous mice and their *Kir6.1^wt/^^wt^* littermate controls are summarized in Figure 1C to J. Compared to WT control vessels, contraction amplitude (C) was reduced by >85% at almost all pressures in *Kir6.1^wt/^^wt^* vessels. Contraction frequency (D) was also reduced by ≥50% in *Kir6.1^wt/^^VM^* vessels at all pressures, with no contractions at the two lowest pressures (except in 1 of 11 vessels at pressure = 1 cmH_2_O), such that FPF (C) was reduced by 85% to 100% in *Kir6.1^wt/^^VM^* vessels, depending on the pressure level. Tone (F) was comparable between *Kir6.1^wt/^^wt^* and *Kir6.1^wt/^^VM^* vessels. In response to 10 μm GLIB (panels G to J), the contraction amplitude was slightly, but not significantly, reduced in *Kir6.1^wt/^^wt^* vessels (compare open symbols in panels C vs. G), and small contractions appeared in *Kir6.1^wt/^^VM^* vessels, but still with substantially lower-than-normal amplitudes (compare closed symbols in panels C vs. G). GLIB enhanced contraction frequency at the other pressures (significantly so in *Kir6.1^wt/^^wt^* vessels, panel (H) and enhanced FPF at most pressures in both groups (panel I). GLIB enhanced tone by ∼2-fold in control vessels and by 3-to-4 fold in *Kir6.1^wt/^^VM^* vessels (panel J). Overall, these experiments reveal substantial impairment in multiple lymphatic contractile parameters in *Kir6.1^wt/^^VM^* mice, with severe reductions in calculated active pump output (FPF) that were partially rescued by GLIB.

Next, we examined lymphatic contractile properties in mice with a CS-associated GoF mutation (A478V) in the SUR2 subunit. Representative recordings of spontaneous contractions in popliteal lymphatic vessels from a heterozygous (*SUR2^wt/AV^*) mouse and a homozygous (*SUR2^AV/AV^*) mouse are shown in Figure 2A and B (the diameter responses of *SUR2^wt/wt^* vessels were similar to those in Figure 1A and are not shown here). The contraction frequency, amplitude, and responses to pressure of the *SUR2^wt/AV^* vessel were similar to those of *SUR2^wt/wt^* littermates and other control vessels, except that the amplitudes at some pressures were slightly reduced. In contrast, the *SUR2^AV/AV^* vessel was slow to develop tone and spontaneous contractions, and the latter were then only ∼50% of those in WT control vessels. Contraction frequency was somewhat irregular and GLIB (10 μm) greatly depressed the contraction amplitude.

A summary of contractile properties of popliteal lymphatics from *SUR2^wt/AV^* heterozygous mice, *SUR2^AV/AV^* homozygous mice, and their *SUR2^wt/wt^* littermate controls is presented inFigure 2C to J. Normalized contraction amplitudes were not significantly different between *SUR2^wt/AV^* and *SUR2^wt/wt^* vessels, but were reduced by 50% to 70% at various pressures in *SUR2^AV/AV^* vessels, compared to *SUR2^wt/wt^* controls (Figure 2C). Contraction frequencies were not significantly different at any pressures between *SUR2^wt/wt^ and SUR2^wt/AV^* vessels (Figure 2D), or between the three genotypes at pressures >2 cmH_2_O, except that there were essentially no contractions at the two lowest pressures in *SUR2^AV/AV^* vessels (only 3 of 12 vessels contracted at 0 or 1 cmH_2_O). FPF was higher in *SUR2^wt/AV^* than *SUR2^wt/wt^* vessels but was significantly reduced (by 50–90%) in *SUR2^AV/AV^* vessels (Figure 2E). Tone was comparable between *SUR2^wt/AV^* and *SUR2^wt/wt^* vessels but, intriguingly, was elevated ∼2-fold in *SUR2^AV/AV^* vessels (Figure 2F). GLIB (10 μm) produced effects on *SUR2^wt/AV^* vessels that were similar to those on *Kir6.1^wt/^^VM^* vessels (Figure 2G to J): lower amplitudes and higher frequencies than control at nearly all pressures, with FPF higher at low pressures, but with no significant effects on tone. GLIB produced minimal effects in *SUR2^AV/AV^* vessels, except for the enhancement of frequency at pressures ≥3 cmH_2_O and a slight elevation in tone. The statistical comparisons between *SUR2^AV/AV^, SUR2^wt/AV^*, and *SUR2^wt/wt^* vessels are given in Table 1. These results suggest that *SUR2^AV/AV^* vessels have a similar lymphatic contractile phenotype as *Kir6.1^wt/^^VM^* vessels, but that *SUR2^wt/AV^* vessels (which reproduce the endogenous genotype in some CS patients) have much milder lymphatic contractile dysfunction as assessed by this protocol.

**Table 1.** Statistical tests for SUR2[A478V] genotypes in Figure 2.

Normalized AMP	Pressure (cmH_2_O)						
	0.5	1	2	3	5	8	10
*SUR2^wt/wt^ vs. SUR2^wt/AV^*	ns	ns	ns	ns	ns	ns	ns
*SUR2^wt/wt^ vs. SUR2^AV/AV^*	**	**	***	****	***	*	ns
*SUR2^wt/AV^ vs. SUR2^AV/AV^*	****	****	***	**	**	*	*
*SUR2^wt/wt^ vs. SUR2^wt/wt^* + GLIB	ns	ns	ns	ns	ns	ns	ns
*SUR2^wt/AV^ vs. SUR2^wt/AV^* + GLIB	ns	**	*	*	ns	ns	ns
*SUR2^AV/AV^ vs. SUR2^AV/AV^* + GLIB	ns	ns	ns	ns	***	**	*
*SUR2^wt/wt^* + GLIB *vs. SUR2^wt/AV^* + GLIB	**	*	*	*	ns	ns	ns
*SUR2^wt/wt^* + GLIB *vs. SUR2^AV/AV^* + GLIB	****	***	***	***	**	ns	ns
*SUR2^wt/AV^* + GLIB *vs. SUR2^AV/AV^* + GLIB	**	**	*	*	**	*	ns

Frequency	Pressure (cmH_2_O)						
	0.5	1	2	3	5	8	10
*SUR2^wt/wt^ vs. SUR2^wt/AV^*	ns	ns	ns	ns	ns	ns	ns
*SUR2^wt/wt^ vs. SUR2^AV/AV^*	ns	ns	ns	ns	ns	ns	ns
*SUR2^wt/AV^ vs. SUR2^AV/AV^*	ns	ns	ns	*	**	*	*
*SUR2^wt/wt^ vs. SUR2^wt/wt^* + GLIB	ns	ns	ns	ns	ns	ns	ns
*SUR2^wt/AV^ vs. SUR2^wt/AV^* + GLIB	***	***	*	**	***	**	**
*SUR2^AV/AV^ vs. SUR2^AV/AV^* + GLIB	ns	ns	**	***	**	*	***
*SUR2^wt/wt^* + GLIB *vs. SUR2^wt/AV^* + GLIB	****	****	**	*	ns	ns	ns
*SUR2^wt/wt^* + GLIB *vs. SUR2^AV/AV^* + GLIB	ns	ns	*	ns	ns	ns	*
*SUR2^wt/AV^* + GLIB *vs. SUR2^AV/AV^* + GLIB	ns	ns	ns	ns	ns	ns	ns

FPF	Pressure (cmH_2_O)						
	0.5	1	2	3	5	8	10
*SUR2^wt/wt^ vs. SUR2^wt/AV^*	ns	ns	ns	ns	ns	ns	ns
*SUR2^wt/wt^ vs. SUR2^AV/AV^*	ns	*	ns	*	*	ns	ns
*SUR2^wt/AV^ vs. SUR2^AV/AV^*	*	*	*	***	**	*	ns
*SUR2^wt/wt^ vs. SUR2^wt/wt^* + GLIB	ns	ns	ns	ns	ns	ns	ns
*SUR2^wt/AV^ vs. SUR2^wt/AV^* + GLIB	**	**	ns	ns	ns	ns	ns
*SUR2^AV/AV^ vs. SUR2^AV/AV^* + GLIB	ns	ns	ns	ns	ns	ns	ns
*SUR2^wt/wt^* + GLIB *vs. SUR2^wt/AV^* + GLIB	***	***	ns	ns	ns	ns	ns
*SUR2^wt/wt^* + GLIB *vs. SUR2^AV/AV^* + GLIB	ns	ns	ns	*	ns	ns	ns
*SUR2^wt/AV^* + GLIB *vs. SUR2^AV/AV^* + GLIB	****	****	****	**	**	ns	ns

Tone	Pressure (cmH_2_O)						
	0.5	1	2	3	5	8	10
*SUR2^wt/wt^ vs. SUR2^wt/AV^*	ns	ns	ns	ns	ns	ns	ns
*SUR2^wt/wt^ vs. SUR2^AV/AV^*	ns	ns	ns	ns	ns	*	*
*SUR2^wt/AV^ vs. SUR2^AV/AV^*	ns	ns	ns	ns	ns	ns	ns
*SUR2^wt/wt^ vs. SUR2^wt/wt^* + GLIB	ns	ns	ns	ns	ns	ns	ns
*SUR2^wt/AV^ vs. SUR2^wt/AV^* + GLIB	ns	ns	ns	ns	ns	ns	ns
*SUR2^AV/AV^ vs. SUR2^AV/AV^* + GLIB	ns	ns	ns	ns	ns	ns	ns
*SUR2^wt/wt^* + GLIB *vs. SUR2^wt/AV^* + GLIB	ns	ns	ns	ns	ns	ns	ns
*SUR2^wt/wt^* + GLIB *vs. SUR2^AV/AV^* + GLIB	ns	ns	ns	ns	ns	ns	ns
*SUR2^wt/AV^* + GLIB *vs. SUR2^AV/AV^* + GLIB	ns	ns	ns	ns	ns	ns	ns

Mixed model ANOVAs with repeated measures (pressure) and Tukey’s multiple comparison tests.

*P < .05; **P < .01; ***P < .001; ^****^P < .0001; ns = not significant.

We also tested the effects of the most common CS-associated mutation, SUR2[R1154Q], on lymphatic contractile properties. This mutation causes a significant GoF in recombinant full length channels.^8,34^ However, when knocked into the murine *ABCC9* locus, the mutation results in alternate splicing that generates a non-functional channel (i.e., a relative loss-of-function, LoF) in mouse cardiac and vascular smooth muscle.^34^ Popliteal lymphatics from *SUR2^RQ/RQ^* mice showed highly variable contractile responses. Contraction amplitude was significantly reduced at almost all pressures in 4 of 7 vessels, but in the other three it was within the normal range of *SUR2^wt/wt^* littermate control vessels. Example recordings of diameter from *SUR2^wt/wt^* and *SUR2^RQ/RQ^* vessels are shown in Figure 3A and B. On average, frequency was slightly elevated in *SUR2^RQ/RQ^* vessels (but significantly so only at one pressure), which is different from the frequency-pressure pattern observed in the other mouse models with CS-associated mutations (Figures 1 and 2), leading to values of FPF and tone not that were significantly different between *SUR2^RQ/RQ^* and *SUR2^wt/wt^* littermate vessels, although there were trends for FPF to be lower at pressures >2 cmH_2_O and tone to be higher at all pressures in *SUR2^RQ/RQ^* vessels. GLIB (10 μm) enhanced the frequency of both *SUR2^RQ/RQ^* and *SUR2^wt/wt^* littermate vessels, reduced the amplitude and enhanced FPF only of *SUR2^wt/wt^* vessels. GLIB did not significantly alter tone in either genotype. These results suggest that *SUR2^RQ/RQ^* vessels have milder and more variable lymphatic contractile dysfunction than either *Kir6.1^wt/^^VM^ or SUR2^AV/AV^* vessels, consistent with the likelihood that overall channel density is reduced (i.e., LoF), even though there is a net GoF due to the presence of the mutation in remaining full-length channels.

Having confirmed the marked reduction of lymphatic contractility associated with CS-associated mutations, we were in position to examine whether lymph transport is affected and/or lymphedema develops in the intact animal. Despite the impairment in lymphatic contractile function documented in *Kir6.1^wt/^^VM^* mice (Figure 1), there were no indications of obvious swelling in the hindlimbs or foot pads of most mice with the most severe CS-associated Kir6.1[V65M] mutation, although 2 of 11 *Kir6.1^wt/^^VM^* mice had a slightly swollen foot pad on one side of their body. We attempted to measure hindlimb volume using plethysmographic methods, as described by other groups,^54,55^ by analysis of the hindlimb weight change over time with the leg placed under a gravitational load (see below); however, the results, even for WT control (C57Bl/6 and *Kir6.1^wt/^^wt^*) mice were highly inconsistent. We also attempted to assess possible increases in subcutaneous water content in the thigh and calf using a commercially available device (MoisturemeterEpiD, Deflin Technologies, Kuopio, Finland), but those results were also highly variable. Thus, we turned to in vivo imaging of popliteal lymphatic dye transport.

Active lymphatic contractions are critical for pumping lymph against adverse pressure gradients that develop in dependent extremities^16^ as well as within lymphatic collector networks where the diastolic pressure rises in successive branches due to intrinsic pumping. Therefore, we imaged popliteal lymphatics in vivo under conditions in which a gravitational load was imposed on the hindlimb. In a previous study,^31^ we found that SM-specific deficiency in connexin-45 resulted in loss of contraction wave entrainment and reduction in conduction speed when popliteal lymphatics were studied ex vivo. Imaging of these vessels in vivo revealed that the dye transport rate was ∼30% lower (although not significantly different) than that of WT vessels when the animal was placed in a horizontal position, but that transport was severely impaired when the animal and hindlimb were oriented in a near-vertical position.^31^ Here, we imaged lymphatics in the hindlimb of *Kir6.1^wt/^^VM^* mice and *Kir6.1^wt/^^wt^* littermate controls under conditions similar to those used in our previous study, except that dye was injected *after* the animal was rotated into the vertical position, rather than before the mouse was rotated. Three different indices of lymph transport were examined: (1) time from injection in the foot to the first appearance of a dye wavefront in one of the two popliteal vessels in the calf; (2) speed of the wavefront; and (3) analysis of the frequency of packets associated with propulsive dye movement. For a useful reference, we also examined the same indices of lymph transport in mice with SM-specific deficiency in the l-type, voltage-gated calcium channel, *Cav1.2* (*Cav1.2^SM-KO^*) in which we have documented a complete lack of active lymphatic contractions.^47,56^

The images in panel (A) of Figure 4 show the appearance and progression (over the course of ∼2.5 min) of the fluorescent dye wavefront in one of the two main popliteal collectors under the skin in the calf region. In this *Kir6.1^wt/^^wt^* mouse, dye first appeared in the imaging field 158 s after injection into the dorsal surface of the foot; it then traveled to the site indicated in the fourth image at an average rate of 107 μm/s. Wavefront tracking beyond this point was seldom possible (unless larger volumes of dye were injected), as both popliteal collectors typically dove deeper into the tissue before eventually entering the popliteal node. Dye subsequently appeared in the saphenous vein of the other leg after 30 to 60 min (not shown). Similar measurements were made on popliteal lymphatics in the legs of *Kir6.1^wt/^^VM^* mice and SM-specific *Cav1.2*-deficient (*Cav1.2^SM-KO^*) mice. An example showing the time of dye appearance and dye wavefront progression in a *Kir6.1^wt/^^VM^* mouse is given in Figure 4B. An example depicting how packet frequency was measured in a vessel in *Kir6.1^wt/^^wt^* mouse is given in Figure 4C, along with representative traces of ROI intensity changes in vessels from *Kir6.1^wt/^^wt^* and *Kir6.1^wt/^^VM^* mice.

Summaries of transport indices for the three genotypes are shown in Figure 4D to F. The average time for wavefront appearance increased significantly, from 14 min in *Kir6.1^wt/^^wt^* vessels, to 24 min in *Kir6.1^wt/^^VM^* vessels, and to 34 min in *Cav1.2^SM-KO^* vessels (Figure 4D). The average wavefront speeds also decreased, albeit not significantly, from 83, to 63, to 50 μm s^−1^ in *Kir6.1^wt/^^wt^, Kir6.1^wt/^^VM^*, and *Cav1.2^SM-KO^* vessels, respectively (Figure 4E). Packet frequency was the most consistently altered index of active pumping, averaging 5 min^−1^ in *Kir6.1^wt/^^wt^* mice, 0.4 min^−1^ in *Kir6.1^wt/^^VM^* mice, and 0 min^−1^ in *Cav1.2^SM-KO^* mice (no packets were observed in any of the latter animals), and the differences between these groups were highly significant (Figure 4F). These indices of active pumping all point to impaired active transport in the intact lymphatic networks of the hindlimbs of *Kir6.1^wt/^^VM^* mice under conditions in which lymph must be propelled against an adverse hydrostatic pressure gradient.

The relatively large variability in indices of in vivo lymphatic transport, and the persistence of lymph transport against a gravitational load, even in *Cav1.2^SM-KO^* vessels (which lack any active lymphatic contractions), are consistent with the presence of other, yet-to-be-defined, factors that contribute to variability in those measurements. Thus, we turned to another ex vivo method to assess the ability of single lymphangions (the fundamental pumping units of lymphatic networks) to propel lymph under an imposed load. In this context, the advantage of an ex vivo method is that it can be performed under defined conditions where Pin and Pout are precisely controlled. When longer, 2-valve segments were studied ex vivo, it became apparent that contraction waves in vessels from mice with CS-associated mutations were unusually slow and not well entrained. We therefore quantified the conduction speed and extent of entrainment in vessels from WT, *Kir6.1^wt/^^VM^, SUR2^wt/AV^*, and *SUR2^AV/AV^* mice by recording videos of spontaneous contractions over the entire segment, at equal Pin and Pout, and then constructing spatiotemporal (ST) maps of the contraction waves. Conduction speed and entrainment index were determined as described in the section “Material and Methods” and a more detailed discussion on the validation and interpretation of these maps was given previously [Figure 1 and Supplementary Figure 2 in ref.^31^]. A representative analysis for a WT (*SUR2^wt/wt^*) vessel is shown in Figure 5A. On the ST map, an entrained contraction wave is represented by a dark, uninterrupted vertical band. The *y*-axis is the position along the vessel and corresponds to the image on the left. The *x*-axis represents time, with each vertical line representing a single video frame. The intensity of the band reflects the degree of constriction (darker = smaller diameter). The nine vertical lines in the top panel of Figure 5A indicate 9 highly entrained contraction waves that initiated at the bottom of the image and conducted along the vessel to the other end. In contrast, the ST maps for a *SUR2^AV/AV^* vessel (Figure 5B) and a *Kir6.1^wt/^^vm^* vessel (Figure 5C) exhibited contraction waves that sometimes initiated at the bottom of the images and sometimes near the middle of the segments, with most of the waves failing to conduct over the entire vessel length. The conduction speeds of the waves were variable but typically much slower than the contraction waves in WT vessels. Additionally, contraction waves in the GoF vessels conducted in either, or both, directions, which was almost never observed in WT vessels. On average, conduction speeds in the GoF vessels were ∼1/10 those in WT vessels (Figure 5C) and the percentages of contraction waves that conducted over the entire vessel length (the “entrainment index”) were only ∼20% of those for WT vessels (Figure 5D). Quantification of the data from *Kir6.1^wt/^^VM^* and *SUR2^wt/AV^* vessels revealed reductions in conduction speed and entrainment deficits in proportion to the relative GoF (Figure 5C and D).

Loss of contraction wave entrainment may not profoundly affect the amplitude or frequency of spontaneous lymphatic contractions when the vessels are studied at equal levels of Pin and Pout, i.e., without any imposed outflow load; however, in the case of connexin-45 deficiency, we previously found that entrained contraction waves are critical for transport against an imposed adverse pressure gradient. Thus, we performed similar tests on popliteal vessels from mice with CS-associated mutations, as shown in the representative recordings in Figure 6A to C. The protocol for the WT vessel was to hold Pin constant at 0.5 cmH_2_O while Pout was slowly raised to 12 cmH_2_O. Initially, when pressures were equal, both valves were open in diastole and sometimes closed transiently in systole; however, when Pout exceeded ∼1 cmH_2_O, the outflow valve closed and remaining closed in diastole, opening only transiently in the systolic phase of each subsequent contraction. This behavior continued until Pout reached ∼9.5 cmH_2_O; above that value the output valve did not open during the subsequent three contractions. The value of Pout at the last successful ejection, minus the value of Pin, was the pump limit for this vessel (9 cmH_2_O). Similar protocols for a *SUR2^AV/AV^* (Figure 6B) and a *Kir6.1^wt/^^VM^* vessel (Figure 6C) revealed pump limits <1 cmH_2_O. For those vessels, Pin was set at 2 cmH_2_O because the vessels did not exhibit any spontaneous contractions at pressures of 0.5 or 1 cmH_2_O. Pump limits for the various mouse genotypes are summarized in Figure 6D and indicate that pumping was severely impacted in lymphatic smooth muscle from mice with CS-associated mutations. The average pump limits for vessels from those mice were each significantly lower than those for WT vessels. Even *SUR2^wt/AV^* vessels, which had contraction amplitudes and frequencies comparable to those of WT vessels in tests at equal levels of Pin and Pout (Figure 2), had a significantly lower pump limit. This finding suggests that the effect of lowered SM excitability on contraction wave entrainment may be an equally significant factor as reduced contraction amplitude in the impairment of propulsive lymphatic contractions.

## Discussion

In the present study, we assessed lymphatic contractile function in mice with CS-associated GoF K_ATP_ mutations engineered into the endogenous loci of *KCNJ8 and* ABCC9. Our results demonstrate that CS-associated mutations in K_ATP_ lead to significant lymphatic contractile dysfunction. Impaired active lymphatic pumping is a likely explanation for the lymphedema reported in CS patients, and would represent a first example of primary lymphedema in which the underlying cause is a lymphatic contractile defect rather than a valve defect or consequence of abnormal vessel or valve development.^57,58^ It would also be the first example of primary lymphedema caused by mutation in an ion channel gene in smooth muscle. With these GoF K_ATP_ mutations, contractile dysfunction manifests ex vivo as a reduction in the amplitude and frequency of spontaneous lymphatic contractions, which together result in reductions in calculated flow by up to 90%, depending on the particular GoF mutation and the pressure at which contractile function is assessed. Reduced excitability of lymphatic muscle in K_ATP_ GoF vessels also leads to loss of contraction wave entrainment, resulting in profound defects in pumping ability under an imposed outflow load. We propose that this combination of contractile defects explains the lymphedema observed in dependent extremities of CS patients. Indeed, a recent report describes a patient originally diagnosed with primary lymphedema who was subsequently found to have a GoF mutation in *ABCC9* and was rediagnosed with CS.^59^

The degree of impairment of contractile function depends on the severity of the molecular consequence of the mutations. The results reported here and those of a previous study,^15^ suggest the following hierarchy of lymphatic contractile dysfunction in K_ATP_ GoF mice: *Kir6.1[GD-QR]* ≈ *Kir6.1^wt/^^VM^* > *SUR2^AV/AV^* > *SUR2^RQ/RQ^* > *SUR2^wt/AV^*. This order correlates with the degree of molecular GoF, and is consistent with the order of severity of cardiovascular defects that were previously noted in mice of the same genotypes.^11,12^ It would be interesting and potentially clinically important to know if the degree of lymphatic dysfunction observed in the present study also corresponds with the severity of lymphedema in CS patients of the same genotypes, but correlation between genotype and human phenotype is currently lacking. The relatively mild lymphatic phenotypes of mice harboring SUR2[A478V] or SUR2[R1154Q] mutations would predict mild or infrequent lymphedema in patients with the homologous mutations. Although SUR2[A478V] has only been identified in one isolated CS individual, SUR2[R1154Q] is the most common CS-associated K_ATP_ mutation, occurring in ∼30% of CS patients.^6^ As recently discussed,^34^ the SUR2[R1154Q] mutation leads to an unexpected alternate splicing in the expressed mouse transcripts that results in marked loss of expressed protein in cardiovascular tissues, counteracting the otherwise marked molecular GoF of R1154Q mutant channels. However, such splicing does not seem to occur in human transcripts with this mutation,^34^ and the marked molecular GoF is consistent with SUR2[R1154Q] subjects experiencing lymphedema. It is possible that lymphatic muscle is more sensitive to the decreased excitability produced by GoF channels, resulting in a net loss of contractile strength in this genotype. Moreover, the apparent lack of Kir6.1/SUR2-dependent K_ATP_ channels in lymphatic endothelial cells^15^ indicates that lymphatic muscle will lack any potential modulatory effects of K_ATP_ GoF in endothelial cells that could be present in CS vascular smooth muscle.^31,60^

Why were we unable to detect any signs of lymphedema in the legs of mice with the most severe CS-associated K_ATP_ GoF mutations (*Kir6.1^wt/^^VM^*)? It is notable that we also failed to observe any hindlimb swelling in *Cav1.2^SM-KO^* mice (with complete absence of active lymphatic contractions) even after 2 h under a gravitational load. Potentially our measurement techniques were not sufficiently sensitive, although no superior ones are currently available. Another explanation is that a *chronic* gravitational load is required in order for lymphedema to develop. We previously presented arguments for why connexin-deficient mice^31^ and other mice with more severe lymphatic system defects, e.g., *Foxc2*- or *Rasa1-*deficiency, which produce lymphatic valve incompetency,^57,58,61^ do not develop detectable peripheral lymphedema in the absence of a chronic gravitational load,^31^ whereas the homologous mutations cause primary lymphedema in a high percentage of human subjects.^62–68^ In many patients, peripheral lymphedema does not develop until after one or more decades of life even though the lymphatic defects are present throughout development and childhood. For example, delayed onset lymphedema is often characteristic of patients with lymphedema distichiasis,^69^ in which *FOXC2* haplodeficiency leads to a reduction in the number and competency of lymphatic valves.^61,70,71^ Likewise, the onset of overt lower limb lymphedema is not obvious in many CS subjects until the teenage years.^6^ Whether that delay is related to puberty, age and/or repetitive exposure to gravitational loads—or other factors—remains to be investigated.

Lymphatic contractile dysfunction might potentially be rescued by inhibitors of the overactive K_ATP_ channels. Treatment with the sulfonylurea inhibitor GLIB was recently shown to reverse many aspects of cardiovascular dysfunction in mouse models with CS-associated mutations.^32^ In that study, implantation of subcutaneous, slow-release pellets to deliver glibenclamide for 4 wks reversed the chronically low arterial pressure and vascular resistance, and reversed the cardiac hypertrophy otherwise observed in *SUR2^wt/AV^* mice; treatment failed, however, to fully reverse cardiac hypertrophy in *Kir6.1^wt/^^VM^* mice, or to reverse certain aspects of the phenotype related to structural remodeling.^32^ A recent clinical study found that GLIB treatment of a human neonate with CS, who presented with a large patent ductus arteriosus, high pulmonary pressures, an enlarged right ventricle, and required continuous positive airway pressure ventilation, potentially due to bronchotracheomalacia, reversed many of those symptoms, with the only major side effect being mild transient hypoglycemia.^72^ In the present study, there was variable rescue of lymphatic contractile function by GLIB, especially in mice with SUR2 GoF mutations. In general, contraction frequency and tone, but not amplitude, were increased, resulting in partial rescue of FPF in *Kir6.1^wt/^^VM^* and *SUR2^wt/AV^* vessels, but essentially no improvement in *SUR2^AV/AV^* vessels. One implication of this finding is that lymphatic function in some, but not all, CS patients might be improved by GLIB treatment, depending on the severity of the K_ATP_ GoF resulting from any specific mutation. In the present study, even the improvement in FPF for *Kir6.1^wt/^^VM^* and *SUR2^wt/AV^* vessels was limited primarily to the lower pressure range. The relevance of this observation remains unclear as mouse popliteal lymphatic pressures in vivo are unknown. It is possible that the concentration of GLIB (10 μm), close to the solubility limit, was not optimal for rescuing lymphatic function. We selected this concentration because it had been previously used in numerous lymphatic studies [see Table 1 in ref.^25^]. However, preliminary tests of other GLIB concentrations (not shown, and made after these protocols were completed) suggested that 1 to 3 μm GLIB might be more effective at reversing depressed contraction frequency without compromising contraction amplitude. Although frequency is determined by the ionic pacemaker while amplitude is determined by pathways controlling excitation–contraction coupling, these two parameters are not independent because a high frequency can limit the time for diastolic filling and thus reduce contraction amplitude.^73^ The unusually high contraction frequency produced by GLIB in some genotypes (approaching 30 contractions min^−1^, Figures 2H and 3H) is ∼50% higher than the maximal frequencies observed under control conditions (∼20 contractions·min^−1^, Figure 2D and 3D) and likely to compromise diastolic filling. Additionally, we found in a recent study that 10 μm GLIB has off-target effects as revealed by decreases in contraction amplitude in SUR^−/−^ vessels and decreases in frequency in Kir6.1^−/−^ vessels^28^; future studies should consider assessing the dose-dependence of GLIB action in this context. It is also possible that chronic expression of GoF K_ATP_ channels results in the upregulation or downregulation of other ion channels or proteins involved in lymphatic pacemaking and/or contractile function.

Finally, the difficulties in consistently quantifying active lymphatic transport in living mice using currently available methods are reflected in the variability of our in vivo measurements compared to our ex vivo assays made under more controlled conditions. For example, ex vivo protocols at equal levels of Pin and Pout showed that *Kir6.1^wt/^^VM^* vessels have substantially impaired contractile activity at pressures <2 cmH_2_O (FPF = 0 ± 0 min^−1^ vs. 2 ± 0.4 min^−1^ for *Kir6.1^wt/^^wt^* vessels and 0 ± 0 min^−1^ vs. 3 ± 0.8 min^−1^ for *SUR2^AV/AV^* vessels; Figures 1E and 2E), whereas in vivo measurements of dye transport show only a 1.5 to 2-fold higher time-to-wavefront appearance for vessels of the same genotypes in vivo (Figure 4D). In addition, there was a near-absence of any dye packets in *Kir6.1^wt/^^VM^* vessels, and complete absence in *Cav1.2^SM-KO^* vessels (Figure 4F), despite the continued appearance of dye, although delayed, in the imaging field (Figure 4D). This suggests that other forces apart from active pumping are complicating this measurement, e.g., passive flow due to the artificial pressure head created by dye injection or subtle external compressive forces (that may have occurred even the absence of any obvious hindlimb movement). A number of other factors might also contribute to the variability of in vivo measurements, such as differences in lymphatic network anatomy from animal-to-animal, such that collectors in the feet of some mice might be closer to the dye injection site. It is not obvious how these factors can be controlled. While our *ex vivo* estimates of lymph flow based on calculated FPF were more consistent, FPF is not the most definitive index of active pumping ability because the frequency term can dominate and mask changes in amplitude that are ineffective for pumping. A better index of active lymph transport is the pump limit,^31,42,43^ assessed in 2-valve lymphatic segments that are forced to pump against a slowly rising outflow pressure. This forces the lymphangion to pump against an adverse pressure gradient, which is the condition in dependent extremities under which lymphedema most often develops in patients. When these tests were performed on vessels from mice with CS-associated mutations, measurements of the lymphangion pump limit revealed that each of the tested genotypes were severely compromised in this respect (≥ 7-fold lower than WT;Figure 6D)—even vessels from *SUR2^wt/AV^* mice in which the traditional estimates of FPF were not significantly different from those of WT vessels (Figure 2). We propose that the loss of contraction wave entrainment and defective pumping are more likely explanations than reduced contraction amplitude/frequency for lymphedema in human patients with the homologous SUR2[A478V] mutation.

In conclusion, *ex vivo* and in vivo assays of lymphatic collector function in mice with CS-associated mutations engineered into genes encoding K_ATP_ channels, indicate that lymphatic contractile dysfunction is a common consequence, potentially explaining lymphedema in CS. As such, it would represent the first documented example of a primary lymphedema in which the underlying cause is a lymphatic contractile defect. The severity of lymphatic contractile dysfunction varies with the nature of the K_ATP_ mutation, with some mutations producing severe defects and other mild defects, and some more likely to be rescued with sulfonylurea treatment than others.

## Data and materials availability

All data needed to evaluate the conclusions in the paper are present in the paper.
